# Estimating historic and recent non-sexual HIV transmission to women in sub-Saharan Africa

**DOI:** 10.3389/fpubh.2026.1876367

**Published:** 2026-09-03

**Authors:** David Gisselquist

**Affiliations:** Independent Researcher, Hershey, PA, United States

**Keywords:** Africa, blood-borne, HIV, nosocomial, women

## Abstract

**Introduction:**

The proportion of HIV infections in sub-Saharan Africa from non-sexual risks has been debated for decades. This article estimates that proportion for women.

**Methods:**

Use information from studies of discordant couples and from national surveys to estimate women’s incidence from sexual transmission and average male-to-female sexual transmission. Compare those estimates to historic increases in women’s infections and to recent incidence.

**Results:**

Based on four studies reporting incidence in women in discordant couples and on information from national surveys, annual HIV incidence in women from sexual transmission as a percent of women’s prevalence as well as annual male-to-female transmission averaged across all infected men can both be (generously) estimated at 10%. In 10 of the 11 countries that reached at least 10% adult prevalence, percentages of pregnant women infected in sentinel surveys or estimated numbers of infected women by country increased at compound annual rates of 17–58% for multi-year periods during 1985–2000; if women’s incidence from sexual transmission was 10% of women’s prevalence, non-sexual transmission accounted for an estimated 41–83% of women’s infections during those periods. Similarly, if 10% of men with unsuppressed viral loads sexually infect one woman per year, an estimated 65% of women’s incident infections in 10 national surveys during 2019–25 were from non-sexual transmission.

**Discussion:**

According to evidence presented here, most HIV infections in women in sub-Saharan have been from blood-borne risks. Investigations of infections suspected from skin-piercing healthcare and cosmetic procedures can find and fix transmitting procedures and warn the public about blood-borne risks.

## Introduction

Based on numbers infected, women in sub-Saharan Africa are the face of the world’s HIV/AIDS epidemic. As of 2024, the estimated 16.3 million infected women (aged ≥15 years) in the region was 1.7 times the 9.6 million infected men in the rest of the world (1). In 2024, an estimated 1.85 women were infected for every infected man in sub-Saharan Africa, while in the rest of the world more than two men were infected for every woman. Infected women outnumber men throughout sub-Saharan Africa except in a handful of island countries with low prevalence. After tests to identify people with HIV infections were developed in the mid-1980s, governments outside sub-Saharan Africa investigated infections suspected to have come from healthcare (nosocomial infections) by testing people attending suspected transmitting facilities in order to find others infected and to find and fix transmitting procedures. During 1986–2026, investigations uncovered and stopped 13 large outbreaks infecting more than 100 to tens of thousands of people (2, 3). Investigations educated both providers and the public about blood-borne risks. Lack of investigations in sub-Saharan Africa along with insufficient education about blood-borne risks has left many people unaware.

National Demographic and Health Surveys (DHS) in sub-Saharan Africa have asked people what they can do to avoid HIV. In 16 countries during 2003–7, only 8.0–43.4% (median 31.9%) of women – and similar percentages of men – said sharing razors or similar instruments was a risk for HIV (among people aware of AIDS but not previously tested to exclude infected people knowing about risks from their own experience) (4). In 2010, Brewer searched the United States (US) National Library of Medicine Gateway, Media/Materials Clearinghouse, and Google for evidence of programs to educate people in the 16 countries about blood-borne risks. The three of 16 countries for which he found no such programs (Eswatini, Lesotho, and Zimbabwe) had the highest HIV prevalence among women and were among the five countries with the lowest percentage of women aware of blood-borne risk (4).

Because governments across sub-Saharan Africa have not investigated suspected blood-borne HIV infections to find and fix transmitting procedures in healthcare and cosmetic services (tattooing, manicures, etc.), continuing blood-borne transmission accounts for an unknown proportion of infections. The contribution of non-sexual transmission to HIV epidemics in sub-Saharan Africa has been considered and debated for more than four decades (5–8). A recent review of studies that sequenced HIV in blood collected from communities in sub-Saharan Africa and looked for similar sequences to identify infections linked by recent transmission suggests non-sexual transmission accounts for a large proportion of the region’s HIV infections (9). This article estimates that proportion for women during 1985–2000 in the 11 countries in which HIV prevalence in adults (aged 15–49 years) reached or exceeded 10% at any time and in 10 national surveys during 2019–2025 (1).

## Methods

A PubMed search and a review of own files found four studies of discordant couples in which most partners were unaware of their HIV infections or risks. Based on women’s incidence in those studies and information from national surveys on HIV infections and recent coitus among married and unmarried men, this article estimates women’s annual incidence from sexual transmission as a percent of women’s prevalence as well as average annual heterosexual transmission from infected men.

Focusing on the 11 countries where adult HIV prevalence reached at least 10%, this article calculates compound annual rates of increase in: (a) the percentages of women found HIV-infected during 1985–90 in sentinel surveys in urban antenatal clinics (10) in four countries; and (b) the estimated numbers of HIV-positive women during 1990–2000 in all 11 countries (1).

For information on women’s HIV incidence in recent years, I searched for national surveys beginning in 2019 or later that measured incidence (using limited antigen avidity assays), prevalence, and viral load suppression. Most such surveys are available from Columbia University’s Population-based HIV Impact Assessment (PHIA) program (11). A search of African government organizations managing national HIV surveys found two more similar surveys during those years (12–14).

To estimate the percentages of women’s infections from non-sexual risks this article compares: the estimated 10% annual increase in women’s HIV prevalence from sexual transmission to annual increases in percentages and numbers of HIV-infected women during 1985–2000; and the estimated 10% average annual rate of male-to-female sexual transmission to percentages of women with incident infections during 2019–25 in 10 national surveys divided by the percentages of men with unsuppressed HIV viral loads.

Because all information used in this study comes from published sources, it did not need ethical review.

## Results

### Estimating male-to-female HIV sexual transmission in sub-Saharan Africa

Women’s HIV incidence in studies of discordant couples in which most partners were unaware of their infections or risks provides a baseline for estimating the rate of women’s HIV incidence from sexual transmission across a population. A PubMed search with the terms (HIV transmission) AND (Africa) AND (discordant couples) on 2 May 2026 returned 173 articles. Twelve of 173 reported studies of incidence in discordant couples in which at least some partners were unaware of their infections or risks; three of 12 clarified that most partners were unaware (15–17). In Mwanza, Tanzania, 1991–95, for example [page 77 in (17)], only a “small number” sought to know their HIV status, and “at follow-up, only 5% of men reported ever using a condom. It therefore seems unlikely that our estimates of HIV transmission were influenced by a change to safer sexual practices…” Similarly, in Masaka, Uganda, 1989–97, less than 10% of adults asked for their test results, and no uninfected partner in discordant couples reported condom use (15). Review of references in a 2003 article on sexual HIV transmission in Africa (7) identified a fourth study reporting women’s incidence in discordant couples in which most partners were unaware of their infections or risks (18). (An earlier assessment of transmission between discordant couples (19) accepted data from a study (20) in which as many as 50% of infected partners were aware of their infections (21); because that study does not meet criteria set here, data from the study are not included in Table 1. Another study used in the earlier analysis does not say how many incident infections in discordant couples were in women (22) and so is not useful here.)

**Table 1:** Women’s HIV incidence in discordant couples in Africa when most partners did not know their HIV status or risk and few used condoms.

Country, community, years	Incident infections	Person-years of follow-up	Infections per 100 person-years
Uganda, Masaka, 1989–97* (15)	22	208	10.6
Uganda, Rakai, 1990–91 (16)	4	43	9.2
Tanzania, Mwanza, 1991–95† (17)	4	40	10.0
Tanzania, Mwanza, 1991–96* (18)	5	60	8.3
Total†	35	351	10.0

^*^Including couples that became discordant during follow-up. † 18 initially concordant negative couples were discordant (15) or concordant p**o**sitive (3) after 16.5 years of follow-up; because the study does not report the sex of partners infected first and second in these couples, table cannot report women’s follow-up time or incidence, if any, in these newly discordant couples. Sources: see references in rows.

The four studies followed discordant couples in Tanzania and Uganda for one or more years during 1989–97 (Table 1). Combining data from the four studies, incidence in women in discordant couples was 10 per 100 person-years (10% per year). Because one study (17) did not report details about incident infections in 18 newly discordant couples (see note to Table 1), the rate of women’s incidence across all four studies combined could be anywhere between 9.6 to 10.4 per hundred person-years depending on the sex of persons infected first and second in the 18 newly discordant couples. On the other hand, incidence in women with HIV-negative husbands in the four studies ranged from 0.15–0.9 (median 0.5) per hundred person-years (15–18). Hence, across the four studies, infected spouses likely infected wives at the rate of *circa* 10% per year. The four studies include men with old and new infections, partners with sexually transmitted infections, circumcised and intact men, and likely some penile-anal sex.

These studies were unethical, following women without warning them about infected partners; I have found no similar studies outside Africa. However, some look-back studies outside Africa estimated HIV transmission from transfusion recipients and hemophiliacs who were unaware they were infected to their partners who were similarly unaware. Findings from such look-back studies generally agree with findings from (unethical) prospective studies in Africa. For example, one study determined that HIV-infected hemophiliac men in the US infected 4 of 24 wives after 12–78 months of unprotected sex (23).

To go from the estimated 10% per year rate of male-to-female sexual transmission in discordant couples in sub-Saharan Africa to the estimated contribution of sexual transmission to women’s incidence across a population, several adjustments must be considered:

- Because the numbers of infected men in the population are less than the numbers of infected women, even if all infected men transmit sexually at the rate of 10% per year (as estimated between discordant couples), annual incidence in women from sexual transmission would be less than 10% of women’s prevalence.
- Many infected men are married to infected women. In national surveys in Kenya in 2003 (24), Mozambique in 2009 (25), South Africa in 2016 (26), and Uganda in 2004–5 (27) a median of 73% (ranging from 45 to 74%) of infected men aged 15–49 years were married; and a median of 56% (ranging from 49 to 74%) of infected and married men (aged 15 to 54–64 years) had infected wives. (These four countries have the largest populations among the 11 countries that reached at least 10% adult HIV prevalence; for the four countries, these surveys are the earliest supervised by the Demographic and Health Services (DHS) Program (28) reporting such data so as to be most relevant to HIV transmission during1985-2000.)
- Unmarried men, on average, have less sex than married men; in the same four surveys (24–27) the median ratio of unmarried to married men reporting coitus in the last 4 weeks was 0.38:1 (ranging from 0.17:1 to 0.57:1).
- Many of men’s non-marital partners, including sex workers, would already be infected.

Considering the above factors, the annual number of new infections in women from sexual transmission as a percent of women’s prevalent infections across a community or population would likely be much less than 10%. And, similarly, the average annual rate of male-to-female sexual transmission across all infected men would likely to be much less than 10% (0.1 per hundred person-years). However, this article uses these unrealistically high 10% estimates to avoid unnecessary controversies over rates of sexual transmission. Evidence outside Africa agrees that these estimates are likely high; e.g., a study in Switzerland used sequencing to estimate that HIV-infected heterosexuals infected a lifetime average of 0.44 others through heterosexual coitus (29).

### Historic increases in women’s infections and continuing incidence

Soon after HIV tests were developed in 1984, African governments with foreign assistance organized studies to assess the region’s epidemics. As of 1988, the WHO estimated that ≥0.5% of adults and children were infected in 11 countries in sub-Saharan Africa (30). In four of the 11 countries (Malawi, Uganda, Zambia, and Zimbabwe), HIV prevalence in adults aged 15–49 years subsequently reached and exceeded 10%. Adult prevalence in the other seven countries estimated to have the worst epidemics in 1988 (Burundi, Central African Republic, Côte d’Ivoire, Democratic Republic of the Congo, Guinea-Bissau, Republic of Congo, and Rwanda) reached 2.4–7.9% in the 1990s before falling back to 0.7–3.2% in 2024 (1).

#### Early increases in HIV infections in antenatal women

The best evidence to see levels and trends of HIV infections in a significant category of women in sub-Saharan Africa before 1990 comes from sentinel surveys that tested women at selected urban antenatal clinics (10). Although percentages of women found infected in urban antenatal clinics generally exceeded percentages of all women infected, trends over time are a good indicator of epidemic trends in the country.

Four of the 11 countries in which adult HIV prevalence subsequently exceeded 10% began testing women in urban antenatal clinics in 1985. In these four countries, compound annual percentage increases in the median percentages of women infected in urban antenatal clinics during 1985–90 ranged from 24% in Uganda to 58% in Malawi (Table 2). In Zimbabwe, the median 18.7% of women infected in four urban antenatal clinics in 1990 is evidence of rapid increases in women’s infections before 1990; but because Zimbabwe’s first antenatal sentinel survey was in 1989, sentinel surveys provide little information about the timing and pace of early increases.

**Table 2:** Increasing percentages of infected women in urban antenatal sentinel sites during 1985–90 in countries in which adult (age 15–49 years) prevalence later reached at least 10%.

Country	Estimated % of children and adults infected, 1988	Median % infected in urban clinics (number of sites)		Annual compound % increase during 1985–90
		1985	1990	
Kenya	<0.5%	2% (1)	8.9% (3)	35%
Malawi	≥1%	2% (1)	19.9% (2)	58%
Uganda	≥1%	10.7% (1)	30.8% (2)	24%
Zambia	≥1%	5% (2)	24.5% (1)	37%
Zimbabwe	≥0.5–0.99%	-	18.7% (4)	-

Sources: estimated 1988 prevalence in adults and children from the WHO (30); percent infected in antenatal clinics from UNAIDS and WHO (10).

Looking beyond medians from multiple clinics, the percentages of women infected in some antenatal clinics were much higher. For example, rural clinics in Rusape and Chiredzi in Zimbabwe reported, respectively, 67 and 70.2% infected in 1995 (10).

#### Increases in women’s infections during 1990–2000

The WHO and later UNAIDS used data from multiple sources, including national surveys, to model each country’s HIV epidemic and thereby to estimate HIV prevalence by year beginning from 1990 (1). In five of the 11 countries in which estimated adult HIV prevalence exceeded 10% for one or more years during 1990–2024, maximum estimated adult prevalence was reached no later than 1997. In those five countries, estimated numbers of infected women increased during 1990–2000 at compound annual rates ranging from 0.8% in Uganda to 9.5% in Kenya (Table 3).

**Table 3:** Increasing numbers of HIV-infected women aged ≥15 years during 1990–2024 in the 11 countries that reached 10% adult prevalence.

Countries that reached ≥10% HIV prevalence in adults (age 15–49 years)			HIV-infected women (‘000 s)			Compound annual % increases in HIV-infections	
Country*	Highest %	Year	1990	2000	2024	1990–2000	2000–24
Uganda	10.9%	1990–01	470	510	930	0.8%	2.5%
Zimbabwe	29.6%	1995	220	490	610	8.3%	0.9%
Kenya	10.1%	1996	360	890	840	9.5%	−0.2%
Zambia	14.0%	1997	520	900	740	5.6%	−0.8%
Malawi	16.0%	1997–98	200	400	840	6.7%	3.1%
Botswana	25.9%	2000	9.5	83	140	24%	2.2%
Namibia	13.8%	2002–03	30	150	220	17%	1.3%
Lesotho	24.9%	2011–13	140	2,100	5,100	31%	3.8%
Eswatini	29.4%	2014	11	130	160	28%	0.9%
Mozambique	12.9%	2016	67	490	1,500	22%	4.8%
South Africa	18.6%	2016–18	2.1	73	130	43%	2.4%

^*^Listed according to the year of their highest adult prevalence. Source: UNAIDS (1).

However, in six of the 11 countries that reached at least 10% adult prevalence, numbers of infected women grew much faster – at compound annual rates of 17–43% – during 1990–2000 (Table 3). Subsequently, numbers of infected women in all 11 countries grew more slowly or even declined during 2000–24 (Table 3).

#### Women’s continuing high incidence

Estimated HIV incidence in sub-Saharan Africa peaked at 2.3 million in the mid-1990s then fell to an estimated 650,000 in 2024. The extension of antiretroviral treatment (ART) to most infected adults over the last several decades not only prolonged lives but also reduced transmission. Most people on ART have suppressed viral loads (<1,000 HIV per milliliter of blood) and almost never transmit through sex (31).

PHIA national surveys in eight countries in sub-Saharan Africa during 2019–2025 reported HIV incidence (with limited antigen avidity assays), prevalence, and percentages with suppressed viral loads (32–39). Governments of Botswana in 2021 and South Africa in 2022 managed similar surveys (12–14). Eight of the 10 surveyed countries are among the 11 in which adult HIV prevalence reached at least 10% (Table 4). In nine surveys, excepting only Mozambique’s in 2021, more than 69% of infected men had suppressed viral loads. Because the 95% confidence intervals for incidence reported from these surveys are wide, this analysis focuses on medians across the 10 countries. The median ratio of HIV incidence in women vs. men is 2.48 (Table 4), which is notably greater than UNAIDS’ estimated 1.85 ratio of infected women to men in sub-Saharan African in 2024; the 2.48 ratio suggests that women have recently accounted for *circa* 71% (≈2.48/[1 + 2.48]) of HIV incidence in regional adults.

**Table 4:** HIV incidence* in women vs. men and rate of sexual transmission required to explain all women’s HIV incidence in ten surveys, 2019–25.

Country, survey years	Ratio of HIV incidence in women vs. men	Rate of male-to-female sexual transmission required to explain all women’s incidence				
		% men with			% annual incidence in women	Required rate per 100 PYs
		HIV	VLS	HIV and no VLS		
		A	B	C = A(1-B)	D	E = D/C
Lesotho, 2019–20 (32)	2.29	17.8%	77.1%	4.07	0.64%	0.17
Zimbabwe, 2019–20 (33)	2.70	10.2%	73.0%	2.75%	0.54%	0.20
Malawi, 2020–21 (34)	2.42	7.1%	85.5%	1.03%	0.29%	0.28
Uganda, 2020–21 (35)	1.90	4.3%	69.8%	1.30%	0.38%	0.29
Botswana, 2021 (12)	∞‡	15.2%	88.1%	1.81%	0.40%	0.22
Eswatini, 2021 (36)	6.53	18.7%	86.1%	2.60%	1.11%	0.43
Mozambique, 2021 (37)	2.54	9.5%	58.8%	3.91%	0.61%	0.16
So Africa, 2022 (13, 14)	1.38	11.3%	77.6%	2.53%	0.69%	0.27
Tanzania, 2022–23 (38)	2.18	3.0%	72.2%	0.83%	0.24%	0.29
Cameroon, 2024–25 (39)	3.33	2.0%	72.0%	0.56%	0.20%	0.36
Medians	2.48		75.1%			0.28

PYs: person-years. So Africa: South Africa. VLS: viral load suppression. * Based on limited antigen avidity assays, viral load, and absence of antiretrovirals. †Aged ≥15 years except 15–64 years in Botswana. ‡ Botswana’s survey reported no incidence in men. Sources: see references in each row.

If all women’s infections in these countries came from sex, men with unsuppressed viral loads would have had to infect, on average, *circa* 0.28 women per year, which is the median across the 10 countries of the percentage of women with incident infections divided by the percentage of men with HIV and unsuppressed viral loads (Table 4, last column).

### Comparing historic increases in women’s infections and ongoing incidence to estimated infections from sexual transmission

In 10 of the 11 countries in which adult HIV prevalence reached at least 10%, percentages of women in urban antenatal clinics or estimated numbers of infected women by country increased at compound annual rates of 17–58% during 1985–90 or 1990–2000 (Tables 2, 3); these rates were 1.7–5.8 times faster than the (generously) estimated 10% annual increase in women’s infections from sexual transmission. With these estimates, non-sexual transmission accounted for an estimated 41% (≈[1.7–1]/1.7) to 83% (≈[5.8–1]/5.8) of women’s incidence in years with high rates of growth in percentages or numbers of infected women. Furthermore, the proportion of women’s infections from non-sexual transmission would have to be even greater to make up for AIDS-related deaths; UNAIDS estimates that AIDS-related adult deaths increased from 2.4% of infected adults in 1990 to 6.8% in 2000 (1).

Similarly, if men’s average rate of heterosexual transmission is 0.1 per year based on observed incidence in women in four studies of discordant couples (Table 1), a median estimate of 64% (≈[0.28–0.1]/0.28) of women’s incidence recognized in 10 surveys during 2019–25 was from non-sexual transmission.

## Discussion

Combining data on women’s incidence from four studies that followed discordant couples in sub-Saharan Africa in which most partners did not know they were infected or at risk, and considering other factors as well, women’s annual incidence as a percentage of women’s prevalence as well as average annual heterosexual transmission from all infected men can be (generously) estimated at 10%.

That 10% rate is far too low to explain the 17–58% compound annual increase in women’s infections during 1985–90 or 1990–2000, or to explain women’s incidence in 10 national surveys during 2019–25. Unless the average annual rate of male-to-female heterosexual transmission is much greater than 10%, non-sexual risks accounted for most women’s infections in the countries and years when Africa’s epidemics grew fastest and continue to account for a large majority of women’s ongoing incidence.

### Women’s non-sexual HIV risks

All women’s non-sexual risks are blood-borne; the risk to get HIV from a breastfeeding infant who was nosocomially infected could be considered an indirect blood-borne risk for women. Research describes many nosocomial risks [injections (6), infusions, dental care, etc.], and there is evidence as well for transmission during skin-piercing cosmetic procedures (tattooing, shaving, etc.). Injection of recreational drugs (IDU) has been a very minor factor; as of 2022, IDU accounted for an estimated 1.3% of HIV incidence in men and women aged 15–49 years in sub-Saharan Africa (40). Paragraphs below note three nosocomial risks peculiar to women.

#### HIV infection associated with antenatal care

Using data from Kenya’s 2003 DHS, Brody and Deuchert reported that women who gave birth and received one or more tetanus vaccinations (common during antenatal care) in the last 5 years were 1.89 (95% confidence interval: 1.03–3.47) times more likely to be HIV-positive compared to other women (adjusted for regional prevalence, women’s age, and number of sexual partners in the last 12 months) (41).

Using 2003–06 data from DHS and AIDS Indicator Surveys, Brewer and colleagues reported similar evidence for women in 11 countries (Burkina Faso, Cameroon, Ethiopia, Ghana, Guinea, Kenya, Lesotho, Malawi, Rwanda, Senegal, and Zimbabwe) (42). Women who gave birth in the last 5 years and received a blood test and/or tetanus vaccination during antenatal care were 1.42 (95% confidence interval: 1.19–1.69) times more likely to be HIV-positive compared to other women (adjusted for country). The study excluded women previously tested for HIV (except in Burkina Faso, where the survey did not ask about prior tests) because those who knew they were infected might seek antenatal care to prevent mother-to-child transmission. (Brewer and colleagues reported partial results at the 2007 conference of the International Society for Sexually Transmitted Diseases Research (43).)

#### Increased HIV risk with Depo-Provera injections for birth control

A 2015 review of 15 studies estimated injections of depot medroxyprogesterone acetate (DMPA, brand name Depo-Provera) for birth control increased women’s risk for HIV infection by 50% compared to women not using hormonal contraception of any kind and by more than 40% compared to women taking combined oral contraceptives (44). Women’s increased risk for HIV infection with DMPA injections may be due not only to the injected drug but also to contaminated injections. In a 2012–16 study in a South African community, women taking injections for birth control acquired HIV infections at the rate of 12% per year compared to 3.7% per year for other women (45). Because that difference far exceeds estimates of increased risk from the injected drug, most of the difference was likely due to contaminated injections.

#### Children with HIV infections from blood exposures infect their mothers

Using evidence from nosocomial outbreaks in Russia and Libya, Little and colleagues estimated that children nosocomially infected with HIV when they were still breastfeeding subsequently infected 40–60% of their mothers, likely through breastfeeding (46). Child-to-mother transmission occurs in sub-Saharan Africa as well, but numbers can only be estimated because nosocomial outbreaks in the region have not been investigated. For example, a 2015 national survey in Mozambique reported 2% of infants aged 6–23 months to be HIV-positive, of which 33% had HIV-negative mothers (47). If 40–60% of children infected in health care in Mozambique had infected their mothers (as in Russia and Libya) before the survey, the 33% of children with HIV-negative mothers reported in the survey would have been 40–60% of children infected in health care. Hence, health care infected an estimated 55% (=0.33/0.6) to 82.5% (=0.33/0.4) of children who were HIV-positive in the survey, of which *circa* 22% (=55–33%) to 49.5% (=82.5–33%) had infected their mothers before the survey. With these estimates, since the survey found 2% of children to be infected, child-to-mother transmission had infected an estimated 0.44 to 0.99% of mothers. At least six other surveys in five countries in sub-Saharan Africa have reported 6–26% of HIV-infected children of various age ranges to have HIV-negative mothers (2).

### Stigma

A small but well-designed survey of clinicians administering ART, 22 adult patients on ART, and 26 caretakers of pediatric patients on ART in 2011 in Maputo, Mozambique, found silence and ignorance about blood-borne HIV transmission. Even when infected patients had uninfected spouses, clinicians rarely told patients their infections might have come from blood-borne transmission (48). No one explained to HIV-negative mothers of infected infants how their children might have been infected. Clinicians’ silence about non-sexual risks in this survey and more generally essentially tells HIV-negative married men and women that infected spouses got HIV from extramarital sexual partners. Fear of being accused of having outside lovers discourages those who know they are infected from warning their possibly uninfected spouses and thereby threatens family stability, health, and children’s welfare.

### Policy implications

To protect patients, the appropriate – proven – response to suspected nosocomial HIV infections is to trace and test others who received treatment at suspected facilities to find the extent of any outbreak and to find and fix transmitting procedures (19). Evidence that blood-borne transmission (including transmission to children who infect breastfeeding mothers) continues to account for a large proportion of women’s infections in sub-Saharan Africa underlines the importance of investigations to protect everyone. Investigations that find and fix transmitting skin-piercing procedures not only protect patients but also educate; investigations that invite people attending specific facilities to come for tests and report how many are infected warn the public about blood-borne risks and combat stigma associating HIV infections with sex. Without investigations, public discussions about blood-borne risks can go some ways towards protecting the public and fighting stigma associating HIV with sex.

## Data Availability

Publicly available datasets were analyzed in this study.
